# Single versus double Hem-o-lok clips to secure the apendiceal stump during laparoscopic appendectomy: a prospective randomized multicentric clinical trial

**DOI:** 10.1007/s00423-024-03281-4

**Published:** 2024-03-08

**Authors:** Ulaş Aday, Erman Çetin, Mehmet Tolga Kafadar, Abdullah Oğuz, Mehmet Veysi Bahadır, Burak Veli Ülger, Ercan Gedik, Sadullah Girgin, Mehmet Yılmaz

**Affiliations:** 1 Department of Gastroenterologycal Surgery, Dicle University School of Medicine, 21280 Sur/Diyarbakır, Turkey; 2 Department of General Surgery, Batman Regional Hospital, Batman, Turkey; 3 Department of General Surgery, Dicle University School of Medicine, Diyarbakır, Turkey

**Keywords:** Laparoscopic appendectomy, Hem-o-lok clips, Apendiceal stump closure, Single clips

## Abstract

**Purpose:**

Polymeric clips (Hem-o-lok ligation system) are now widely used to securing the base of the appendix during laparoscopic appendectomy. Studies comparing the use of single or double hem-o-lok clips are limited. The aim of this study was to compare the reliability of a single hem-o-lok clips with a double hem-o-lok clips for closure of an appendiceal stump.

**Methods:**

This prospective randomized study includes patients from two centers who underwent laparoscopic appendectomy with the diagnosis of appendicitis between September 2020 and March 2023. Demographic, operative and clinical outcomes of the use of single or double hem-o-lok clips for closure of the appendiceal stump were compared. Factors affecting long postoperative hospital stay were investigated using univariate and multivariate analyzes.

**Results:**

One hundred forty two (48.3%) patients in the single hem-o-lok arm and 152 (51.7%) patients in the double hem-o-lok arm were included in the analysis.The shortest operative time was noted in the single hem-o-lok group (52.1 ± 19.9 versus 61.6 ± 24.9 min, *p* < 0.001). The median hospital stay was 1 day (range 1–10) in the single hem-o-lok group and 1 day (range 1–12) in the double hem-o-lok group, and was shorter in the single hem-o-lok arm (1.61 ± 1.56 vs 1.84 ± 1.69, *p* = 0.019). Based on multivariate analysis, drain placement was identified as an independent predictive factor for long hospital stay.

**Conclusions:**

The use of single hem-o-lok clips for appendiceal stump closure during laparoscopic appendectomy is safe and effective.

Trial registration NCT04387370 (http://www.clinicaltrials.gov).

## Introduction

Acute appendicitis is among the most common causes of acute abdomen and laparotomy in young patients admitted to the emergency department with abdominal pain. Laparoscopic appendectomy (LA) has become the gold standard in many centers nowadays due to the advantages it provides in the treatment of acute appendicitis [1, 2]. Safe closure of the appendix stump during LA is important to prevent undesirable clinical complications. Moreover, reducing the cost, shortening the surgical time can reduce the clinical burden for this frequently performed surgical procedure [3–5]. Various technical modifications of stump closure during LA are currently available—closure with a clip, closure using an endoloop or staples [6–10].

In recent years, an alternative method has been reported and involves the use of a nonabsorbable polymer locking ligation system (Hem-o-lok) to secure the appendiceal stump [11, 12]. The hem-o-lok system is low cost, easy to use for the surgeon, and offers safe performance. Hem-o-lok clips are not safe for use in complicated appendicitis such as perforation or inflammation of the base of the appendix, or when the appendix lumen diameter is large [13, 14]. Studies comparing the hem-o-lok clips with other methods have been reported [7–11, 14, 15] but we have not observed studies of single and double hem-o-lok application. The aim of this study was to compare the effect of two different methods of appendiceal stump closure (single or double hem-o-lok clips) on clinical outcomes during laparoscopic appendectomy. The secondary aim of the study was to determine the factors affecting prolonged hospital stay.

## Patients and methods

### Study design and patients

This was a prospective randomized clinical trial designed to evaluate the clinical outcomes of appendix stump closure with single or double hem-o-lok clips in patients undergoing LA. The study was completed at the Department of General Surgery, Dicle University School of Medicine (Diyarbakır, Turkey) and the Department of General Surgery, Batman Regional Hospital (Batman, Turkey). All patients with clinical signs of acute appendicitis were evaluated for study eligibility between September 2020 and March 2023 in two centers. The study was approved by the Dicle University School of Medicine Ethics Committee (approval number: 09/152/2020). Written informed consent was obtained from all patients included in the study and the standards of the Declaration of Helsinki (1964) were followed. The trial was registered at http://www.clinicaltrials.gov (NCT04387370).

**Fig. 1:**
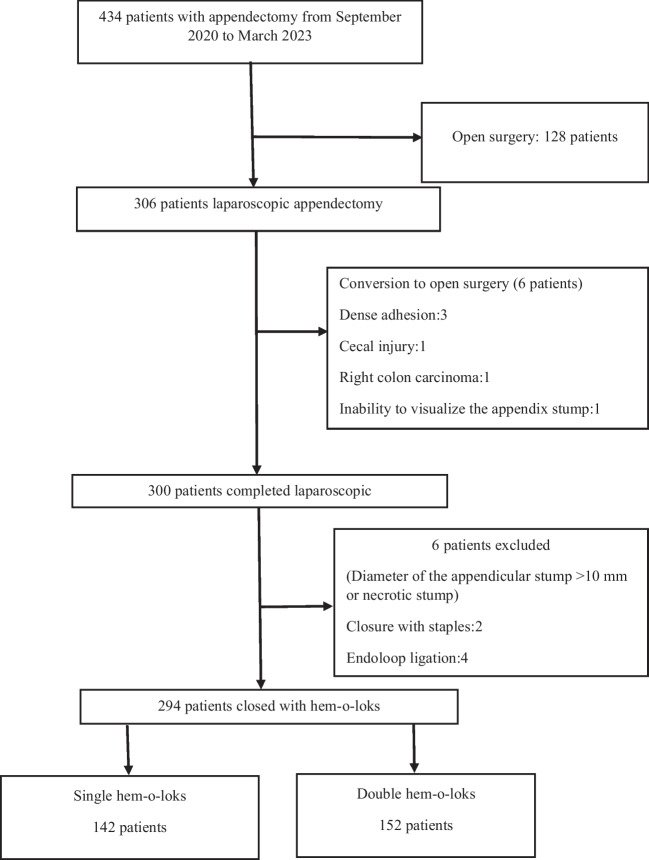
Flow chart of the study

Inclusion criteria: patients diagnosed with acute appendicitis in the surgical emergency department, age ≥ 18 years, and laparoscopic completion of the surgical procedure. Exclusion criteria were: difficulty in dissection and conversion to open surgery for other conditions, necrosis at the stump of the appendix, presence of a lumen wider than the width of the hem-o-lok clip (> 10 mm), and age < 18 years. During the study period, each study subject was randomized preoperatively by the following method: single hem-o-lok if operated on odd days of the month, and double hem-o-lok if operated on even days of the month. All abdominal emergency surgical procedures including trauma are performed in both centers. During the study period, open appendectomy was performed when laparoscopic equipment and supplies were inadequate and these patients were excluded from the study (Fig. 1). Clinical outcomes for single and double clips use are the primary outcome measures of the study and factors affecting prolonged hospital stay are secondary outcome measures.

### Surgical procedure

**Fig. 2:**
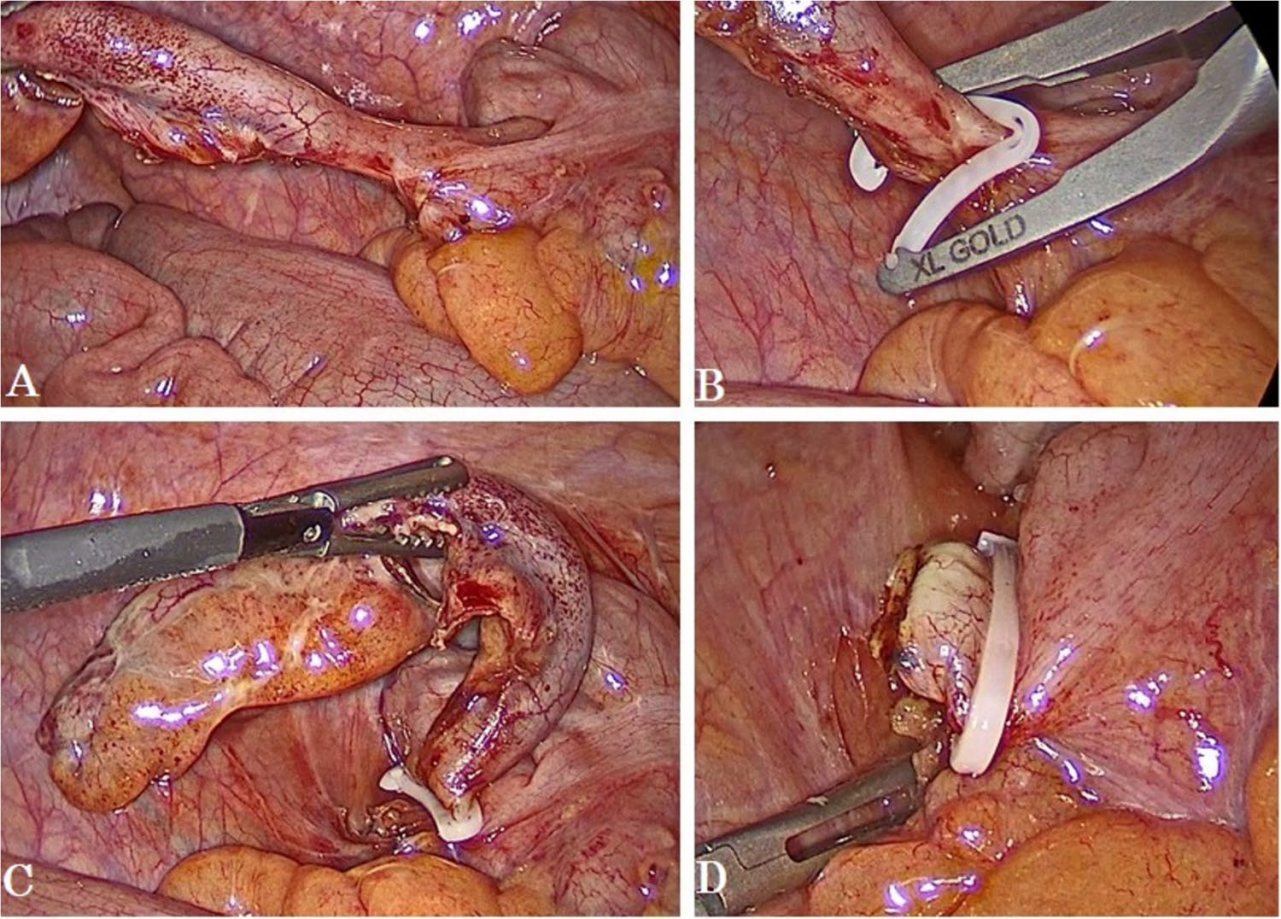
Intraoperative steps of single hem-o-lok clips application

**Fig. 3:**
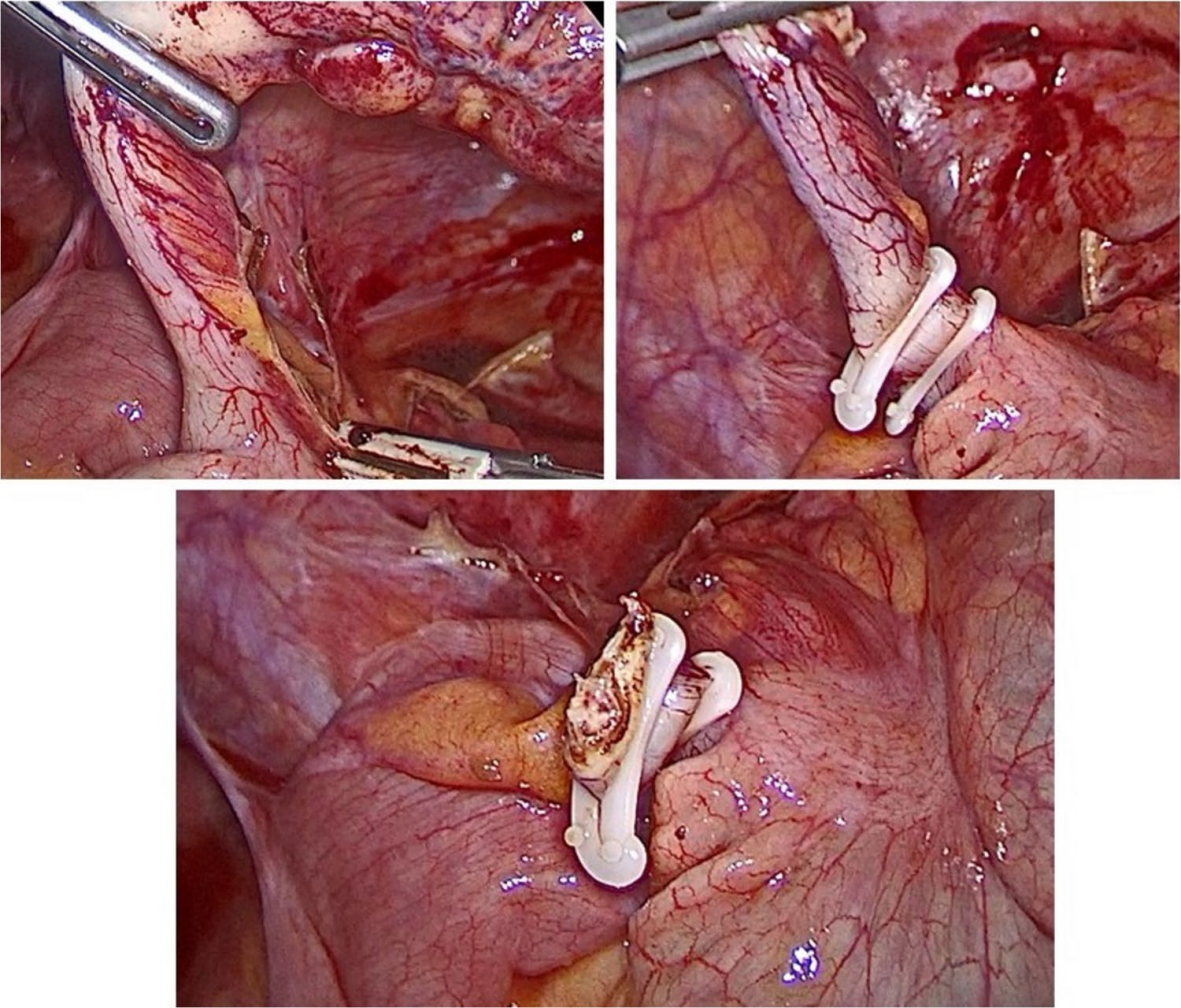
Intraoperative steps of double hem-o-lok clips application

LA was performed by surgeons experienced in advanced laparoscopic surgery in all patients included in the study. The surgical procedure was performed under general anesthesia; the patient was positioned supine, Trendelenburg position, angled to the left. Three port techniques (a 10 mm camera port site in the supraumbilical region, a 12 mm port in the left lower quadrant or upper right quadrant and a 5 mm port in the suprapubic region) were used. For postoperative analgesia, diluted long-acting local anesthetic agent was applied to the port sites. After observation of the abdominal cavity for the presence of additional pathologies, the mesoappendix was divided with an energy device. After preparation of the appendiceal base, either single or double hem-o-lok clips, size XL (Hem-o-lok, Weck Closure Systems, Research Triangle Park, NC, USA) was placed on the base of the appendix by a special applier for the hem-o-lok clip, and another clip was used on the distal part which would be removed stump (Figs. 2 and 3). Finally, the locked status of the clip was checked. Drain placement (in the presence of abscess cavity or purulent peritonitis) was left to the surgeon's decision.

### Data collection and f ollow-up

Demographic and clinical (age, gender, BMI, ASA classification comorbid conditions, time between symptom onset and surgery, etc.) data were collected prospectively. Intraoperative complications, additional pathology status, drain placemen, and duration of surgery were recorded on the registration form. In intra-operative observation, appendiceal pathology was divided into 4 categories; normal findings, suppurative (catarrhal or phlegmonous), gangrenous and perforated. The postoperative surgical complications were graded according to the Clavien-Dindo Classification [16]. Postoperative complications within 30 days, reasons for readmission to the hospital, and final pathologic results were recorded. Hospital length of stay beyond 3 days was considered as prolonged and univariate and then multivariate logistic regression analysis was performed to determine the risk factors affecting the length of hospitalization.

### Statistical analysis

Data were analyzed using IBM SPSS Statistics for Windows ver. 21.0 (IBM Corp., Armonk, NY, USA). Qualitative data obtained in the study were expressed as frequency (percentage), quantitative data as mean ± standard deviation and median (minumum-maximum). Compliance of quantitative data with normal distribution was analyzed by Shapiro Wilk test. Chi-square test was used to analyze categorical data and Mann–Whitney U test was used to analyze quantitative data. Univariate and then multivariate logistic regression analysis was performed to determine the risk factors affecting the length of hospitalization. *P* < 0.05 was taken as the significance level.

## Results

During the study period, 434 patients underwent appendectomy, 128 open and 306 laparoscopic. Six patients were excluded from the study due to conversion to open surgery (dense adhesion, cecal injuri, right colon carcinoma, and inability to detect the stump) 6 patients were excluded due to necrosis at the base of the appendix and closure by other methods due to the lumen diameter being larger than the diameter of the hem-o-lok (Fig. 1). A total of 142 (48.3%) patients in the single hem-o-lok arm and 152 (51.7%) patients in the double hem-o-lok arm were included in the final analysis. First 30-day follow-up was complete in all patients, and no mortality was observed in each group.

The median age of the participants was 30 years (18–80). 123 (41.8%) were female and male patients were the majority in both groups (*p* = 0.042). Demographic, clinical and operative findings of both groups are presented in Table 1. The time from symptom onset to surgery was similar in the single and double clips groups and was 23 (4–154) and 24 (4–168) hours, respectively (*p* = 0.134). Intraoperative findings revealed normal appendix in 5 (3.5%) and 7 (4.6%) patients in the single and double hem-o-lok groups, respectively, while perforation was detected in 13 (9.2%) and 21 (13.8%) patients, respectively. The operative time was 52.1 ± 19.9 min. in the single hem-o-lok arm and 61.6 ± 24.9 min. in the double hem-o-lok arm and the difference was statistically significant (*p* < 0.001).

**Table 1:** Characteristics of the single and double hem-o-lok clips groups

	Single (*n* = 142)	Double (*n* = 152)	Total (*n* = 294)	*P*-value
Age (mean ± SD)	33.05 ± 12.3	33.03 ± 11.6	33.04 ± 11.9	0.707
Age, years*	30 (18–74)	30 (18–80)	30 (18–80)	
Gender, *n* (%)				
Female	68 (47.9)	55 (36.2)	123 (41.8)	0.042
Male	74 (52.1)	97 (63.8)	171 (58.2)	
BMI (kg/m^2^, mean ± SD)	25.5 ± 3.2	25.9 ± 3.1	25.7 ± 3.1	0.706
Comorbidity, *n* (%)				
Diabetes mellitus	3 (2.1)	2 (1.3)	5 (1.7)	0.675
Coronary artery disease	4 (2.8)	2 (1.3)	6 (2.0)	0.434
Hypertension	7 (4.9)	7 (4.6)	14 (4.8)	1.000
Pulmonary disease	0 (0.0)	3 (2.0)	3 (1.0)	0.248
Other	5 (3.5)	6 (3.9)	11 (3.7)	1.000
ASA classifcation, *n* (%)				
< III	140 (98.6)	149 (98.0)	289 (98.3)	1.000
≥ III	2 (1.4)	3 (2.0)	5 (1.7)	
Duration of symptoms, hours *	23 (4–154)	24 (6–168)	24 (6–168)	0.134
Operative diagnosis, *n* (%)				
Normal appendix	5 (3.5)	7 (4.6)	12 (4.1)	
Catarrhal or phlegmonous	112 (78.9)	118 (77.6)	230 (78.2)	0.258
Gangrenous	12 (8.5)	6 (3.9)	18 (6.1)	
Perforated	13 (9.2)	21 (13.8)	34 (1.6)	
Intraoperative complication, *n* (%)	1 (0.7)	2 (1.3)	3 (1.0)	1.000
Gynecological pathology, *n* (%)	6 (4.2)	8 (5.3)	14 (4.8)	0.914
Other pathology, *n* (%)	3 (2.1)	3 (2.0)	6 (2.0)	0.914
Drain placement, *n* (%)	23 (16.2)	33 (21.7)	56 (19.0)	0.229
Operative time (minutes, mean ± SD)	52.1 ± 19.9	61.6 ± 24.9	56.9 ± 23.1	< 0.001
Operative time, minutes*	45 (18–120)	60 (25–180)	50 (18–180)	

*ASA,* American Society of Anaesthesiologist; *BMI*, body mass index; *SD*, standard deviation;

^*^Median (range)

The median hospital stay was 1 day (range 1–10) in the single hem-o-lok group and 1 day (range 1–12) in the double hem-o-lok group, and was shorter in the single hem-o-lok arm (1.61 ± 1.56 vs 1.84 ± 1.69 days, *p* = 0.019). Major complications (Clavien-Dindo classification ≥ III) requiring invasive intervention developed in 3 patients each in the single and double clips groups (Table 2). None of the patients developed stump leakage and clinical conditions requiring relaparatomy. Pathologically, the appendix was reported as normal in 8 (2.7%) patients, mucinous neoplasia in one patient and tuberculous in one patient. In the univariate and multivariate analysis performed to determine the risk factors for prolonged hospital stay, it was observed that the use of single or double hem-o-lok clips was not a risk factor, whereas only intra-abdominal drain placement was an independent risk factor in the multivariate analysis (*p* < 0.001, Table 3).

**Table 2:** Postoperative clinical and pathological outcomes

	Single (*n* = 142)	Double (*n* = 152)	Total (*n* = 294)	*P*-value
LOS*	1 (1–10)	1 (1–12)	1 (1–12)	0.019
LOS (mean ± SD)	1.61 ± 1.56	1.84 ± 1.69	1.73 ± 1.63	
Clavien-Dindo classification, *n* (%)				
< III	139 (97.9)	149 (98.0)	288 (98.0)	1.000
≥ III	3 (2.1)	3 (2.0)	6 (2.0)	
Readmission to hospital, *n* (%)	1 (0.7)	3 (2.0)	4 (1.4)	0.623
Pathologic diagnosis, *n* (%)				0.496
No pathology	3 (2.1)	5 (3.3)	8 (2.7)	
Appendicitis	138 (97.2)	146 (96.1)	284 (96.6)	
Neoplasia	1 (0.7)	0 (0.0)	1 (0.3)	
Tuberculosis	0 (0.0)	1 (0.7)	1 (0.3)	

*LOS*, length of stay

^*^Median (range)

**Table 3:** Univariate and multivarite analysis for long (> 3 day) postoperative hospital stay

Variable	Univariate		Multivariate	
	OR (95% CI)	*P* value	OR (95% CI)	*P* value
Age ≥ 65	7.0 (1.36–36)	0.020	16.33 (0.36–771.6)	0.156
Male sex	0.35 (0.17–0.70)	0.358		
ASA grade ≥ III	10.54 (1.70–65.25)	0.011	0.04 (0.0–4.04)	0.173
Diabetes mellitus	10.54 (1.70–65.25)	0.011	5.59 (0.02–1300)	0.536
Coronary artery disease	7.0 (1.36–36.01)	0.020	1.09 (0.01–122)	0.971
Pulmonary disease	13.73 (1.21–155.1)	0.034	22.1 (0.312–1574)	0.154
Duration of symptoms, ≥ 48 h	43.24 (5.87–26.45)	< 0.001	2.54 (0.57–11.3)	0.221
Operative diagnosis, perforated	16.25 (2.39–90.07)	0.001	9.57 (0.43–213)	0.154
Operative time, ≥ 60 min	7.32 (3.11–17.23)	< 0.001	1.75 (0.37–8.29)	0.479
Gynecological pathology	4.20 (1.32–13.31)	0.015	1.64 (0.17–15.6)	0.665
Stump closure, double hem-o-lok	1.80 (0.89–3.62)	0.099		
Drain placement	229.7 (51.39–1027)	< 0.001	88.5 (12.7–616)	< 0.001

## Discussion

Nowadays, LA has become the method of first choice in the treatment of both complicated and noncomplicated acute appendicitis when laparoscopic equipment and an experienced team are available. LA increases surgical time and operative cost compared to open appendectomy, but offers significant advantages in terms of less postoperative pain, lower incidence of surgical site infection, shorter hospital stay, earlier return to work, overall costs and better quality of life scores. The other contribution of laparoscopy is that it allows the diagnosis and treatment of concomitant pathologies [7, 17, 18]. A key step during LA is securing the appendiceal stump. Different methods have been described to close the appendix stump. There is no universal consensus on any one method and it is observed that different methods are preferred in the literatüre [19].

There are two important factors for the widespread acceptance of new surgical procedures: safety and ease. Current evidence suggests that polymeric clips are a safe and cost-effective method for stump closure during LA [12]. Studies comparing the use of single hem-o-lok clips [8, 9, 20, 21] and double hem-o-lok clips [7, 10, 11, 15, 22] with other methods are available. We have not observed a comparative study of appendicecal stump closure with single hem-o-lok versus double hem-o-lok clips. In our study, we observed that the use of a single hem-o-lok clips for closure of the appendicecal stump in LA shortened the surgical time and hospital stay, and the postoperative complication rates were similar to those of double hem-o-lok clips. We believe that the use of a single hem-o-lok will be the preferred method in this common surgical procedure as it saves time and reduces the number of clips. The use of single or double polymeric clips was not a risk factor for prolonged hospital stay in both univariate and multivariate analysis. In multivariate analysis, only the use of drain was determined as an independent risk factor. In our clinical practice, in the presence of a localized abscess, local (purulent material in the periappendicular area or pelvis) or diffuse peritonitis, a drain is placed and postoperative antibiotherapy is continued. Draining is often performed in complicated appendicitis and is associated with a severe clinical condition with prolonged hospitalization. Nevertheless, a selective use of drains in LA may improve the length of hospitalization [23, 24].

In the current study, postoperative major complications (Clavien-Dindo ≥ III) occurred in 3 patients in each group, all of these complications were intraabdominal infected collections and were resolved by percutaneous drainage and antibiotherapy. There were no complications requiring relaparatomy and mortality. Studies indicate that 1.2–6% of patients require re-intervention after LA [1, 4, 10]. In the study by Soll and colleagues [14], intra-abdominal abscess was observed in 4% of the endoloop group and in 1% of the hem-o-lok group and they found that the use of hem-o-lok reduced the incidence of abscess development. It is believed that double clipping may result in a necrotic stump, increasing the frequency of bacterial infection and adhesions [20]. Necrosis of the appendiceal stump, large diameter and excessive inflammation may limit the use of polymeric clips. Endostapler will be the safe option in cases where there is insufficient stump length for clipping due to necrosis at the base and the diameter is over 10 mm [9, 12].

Nevertheless, our study has several limitations. When the sample size of the study was not calculated by a statistician before the study, the sample size may not be sufficient. During the study period, some patients were treated with open surgery due to lack of equipment, which resulted in not all patients being included in the study. Another limitation is that the use of drains was decided by the surgeon and different disease stages were evaluated together. Lastly, the assignment of patients according to the date of surgery weakens the effective randomisation. However, to our knowledge, this is the first prospective study comparing single and double polymeric clip closure of the appendiceal stump during LA.

## Conclusion

In conclusion, in this prospective study, single hem-o-lok clips is a safe method for appendicecal stump closure in laparoscopic appendectomy. Considering its advantages such as shortening the operating time and reducing the number of clips, it may be the first choice in appendiceal stump closure in laparoscopic appendectomy. We believe that the selective use of drains will shorten the length of hospital stay.

## Data Availability

The data of this study can be obtained from the authors with permission from Dicle University School of Medicine and Batman Regional Hospital.
